# Supplementary material for: NAD+ regulates nucleotide metabolism and genomic DNA replication
Source: Nat Cell Biol. 2023 Nov 13;25(12):1774–86. doi: 10.1038/s41556-023-01280-z (PMC10709141; doi:10.1038/s41556-023-01280-z)
Supplement: Supplementary file 1 — Reporting Summary [file 41556_2023_1280_MOESM1_ESM.pdf]

nature portfolio

Corresponding author(s): Jiri Bartek
Apolinar Maya-Mendoza

Last updated by author(s): Oct 6, 2023

Reporting Summary

Nature Portfolio wishes to improve the reproducibility of the work that we publish. This form provides structure for consistency and transparency in reporting. For further information on Nature Portfolio policies, see our Editorial Policies and the Editorial Policy Checklist.

Statistics

For all statistical analyses, confirm that the following items are present in the figure legend, table legend, main text, or Methods section.

n/a	Confirmed	
☐	☒	The exact sample size (*n*) for each experimental group/condition, given as a discrete number and unit of measurement
☐	☒	A statement on whether measurements were taken from distinct samples or whether the same sample was measured repeatedly
☐	☒	The statistical test(s) used AND whether they are one- or two-sided<br>*Only common tests should be described solely by name; describe more complex techniques in the Methods section.*
☒	☐	A description of all covariates tested
☐	☒	A description of any assumptions or corrections, such as tests of normality and adjustment for multiple comparisons
☐	☒	A full description of the statistical parameters including central tendency (e.g. means) or other basic estimates (e.g. regression coefficient) AND variation (e.g. standard deviation) or associated estimates of uncertainty (e.g. confidence intervals)
☐	☒	For null hypothesis testing, the test statistic (e.g. *F*, *t*, *r*) with confidence intervals, effect sizes, degrees of freedom and *P* value noted<br>*Give P values as exact values whenever suitable.*
☒	☐	For Bayesian analysis, information on the choice of priors and Markov chain Monte Carlo settings
☒	☐	For hierarchical and complex designs, identification of the appropriate level for tests and full reporting of outcomes
☒	☐	Estimates of effect sizes (e.g. Cohen's *d*, Pearson's *r*), indicating how they were calculated

Our web collection on statistics for biologists contains articles on many of the points above.

Software and code

Policy information about availability of computer code

Data collection	Zeiss ZEN (v3.2 blue edition) software, for LSM microscope image acquisition<br>Olympus scanR 3.3 software, for QIBC image acquisition<br>BD FACSuite V1.0.6 software, for flow cytometry data acquisition<br>MinKNOW (v20.06.4), for RNAseq data acquisition
Data analysis	ZEN lite (v2.6 blue edition) software, analysis of LSM-acquired micrographs<br>FlowJo_V10, flow cytometry analysis<br>R (v4.0.4), numerical and statistical analysis and final figure assembly<br>Guppy (v4.0.15, ONT), basecalling<br>Porechop (v0.2.4), demultiplexing and trimming for adapters<br>NanoFilt (v2.6.0), filtering<br>nanoQC (v0.9.4), quality inspections<br>NanoPlot (v1.29.0), quality inspections<br>Minimap2 (v2.17), genome alignment<br>Samtools (v1.9), alignment sorting, BAM conversion, and genome indexing<br>StringTie2 (v2.1.4), transcript assembly<br>GffCompare (v0.12.1), comparison and annotation of the assembled transcriptome<br>GffRead (v0.12.3), realignment to the assembled transcriptome<br>Salmon (v1.4.0), transcriptome alignments quantification<br>DESeq2 (v1.30.1), RNA-seq differential expression analysis<br>Tximport (v1.18.0), transcript count import and gene level summarization

Pheatmap (v1.0.12), heatmap visualizations
Compound Discoverer (3.1), metabolomics compound identification
STRING (v11-0b, webtool), functional enrichment analysis
ScanR analysis software (v3.2.0), analysis of data from ScanR QIBC acquisition

For manuscripts utilizing custom algorithms or software that are central to the research but not yet described in published literature, software must be made available to editors and reviewers. We strongly encourage code deposition in a community repository (e.g. GitHub). See the Nature Portfolio guidelines for submitting code & software for further information.

Data

Policy information about availability of data

All manuscripts must include a data availability statement. This statement should provide the following information, where applicable:

- Accession codes, unique identifiers, or web links for publicly available datasets
- A description of any restrictions on data availability
- For clinical datasets or third party data, please ensure that the statement adheres to our policy

Data availability
RNA–seq data reported in this study have been deposited in the European Nucleotide Archive (ENA) with accession PRJEB64552. Differential expression analyses are provided in Source Data Files. Metabolomics data are provided as Supplementary Tables. Other numerical source data have been provided in Source Data Files. All other data supporting the findings of this study are available from the corresponding author on request.

Human research participants

Policy information about studies involving human research participants and Sex and Gender in Research.

Reporting on sex and gender	This study does not involve human research participants.
Population characteristics	N/A
Recruitment	N/A
Ethics oversight	N/A

Note that full information on the approval of the study protocol must also be provided in the manuscript.

Field-specific reporting

Please select the one below that is the best fit for your research. If you are not sure, read the appropriate sections before making your selection.

☒ Life sciences ☐ Behavioural & social sciences ☐ Ecological, evolutionary & environmental sciences

For a reference copy of the document with all sections, see nature.com/documents/nr-reporting-summary-flat.pdf

Life sciences study design

All studies must disclose on these points even when the disclosure is negative.

Sample size	No sample-size calculation were performed. The sample size was determined to be sufficient, taking into account the substantial and consistent differences observed between the groups. For DNA fiber experiments, if equivalent experiments were not different statistically (p value of t-test <0.001), the total number of DNA fibers from all experiments is shown. Data reported from high-content microscopy experiments are from 2. 3 or 4 biological replicates as are indicated in the figure legends.
Data exclusions	No exclusion was applied.
Replication	Reported results were tested and confirmed in at least two independent experiments (i.e. minimum two biological replicates).
Randomization	Our experimental designs did not permit randomization to be applied.
Blinding	Wherever possible, the investigators were blinded for data acquisition. Samples were labeled only by sequential numbers during their preparation before and, in the majority of cases, during data collection, image acquisition and metabolite analysis.

Reporting for specific materials, systems and methods

March 2021

We require information from authors about some types of materials, experimental systems and methods used in many studies. Here, indicate whether each material, system or method listed is relevant to your study. If you are not sure if a list item applies to your research, read the appropriate section before selecting a response.

Materials & experimental systems

n/a	Involved in the study
☐	☒ Antibodies
☐	☒ Eukaryotic cell lines
☒	☐ Palaeontology and archaeology
☒	☐ Animals and other organisms
☒	☐ Clinical data
☒	☐ Dual use research of concern

Methods

n/a	Involved in the study
☒	☐ ChIP-seq
☐	☒ Flow cytometry
☒	☐ MRI-based neuroimaging

Antibodies

Antibodies used

Rat anti-BrdU antibody, Serotec, OBT0030 (diluted 1:500)
Mouse anti-BrdU antibody, Becton Dickinson, 347580 (diluted 1:500)
DyLight 550, Thermo Fisher Scientific, SA5-10019 (diluted 1:400)
Alexa Fluor 488, Invitrogen, A21202 (DNA fibres, diluted 1:400)
Alexa Fluor 488, Invitrogen, A11029 (QIBC, diluted 1:1,000)
Fibrillarin, Abcam, ab5821 (diluted 1:500)
p-CHK1 S317, Cell Signaling, 2344 (diluted 1:250)
CHK1, Santa Cruz, sc-8408 (diluted 1:100)
p-CHK2 T68, Abcam, ab32148 (diluted 1:200)
CHK2, Abcam, ab109413 (diluted 1:20,000)
p-H2A.X S139 (γH2AX), Abcam, ab22551 (diluted 1:500)
β-Actin, Sigma, A1978 (diluted 1:5,000)
p-AMPKα T172, Cell Signaling, 2535 (diluted 1:500)
AMPKα, Cell Signaling, 2603 (diluted 1:500)
p-mTOR S2481, Cell Signaling, 2974 (diluted 1:250)
mTOR, Cell Signaling, 2972 (diluted 1:250)
α-Tubulin, GeneTex, GTX628802 (diluted 1:5,000)
SLC25A51/MCART1, Abcam, ab237054/CUSABIO CSB-PA875649LA01HU (diluted 1:500)
Vinculin, Sigma, V9131 (diluted 1:50,000-100,000)
VDAC, Cell Signaling, 4661 (diluted 1:500)
COX IV/CIV, Cell Signaling, 11967 (diluted 1:500)
OPA1, Abcam, ab157457 (diluted 1:1,000)
ATPB, Abcam, ab14730 (diluted 1:50)
Horseradish peroxidase-conjugated anti-rabbit secondary antibody (PI-1000, Vector Laboratories, diluted 1:10,000)
Horseradish peroxidase-conjugated anti-mouse secondary antibody (PI-2000, Vector Laboratories, diluted 1:10,000)

Validation

Rat anti-BrdU antibody, validated by the company and cited in 284 research publications
Mouse anti-BrdU antibody, monoclonal antibody originally described in Science 1982; 218:474, validated by the company and cited in 1036 research publications
DyLight 550, validated by the company
Alexa Fluor 488 (Invitrogen, A21202), validated by the company
Alexa Fluor 488 (Invitrogen, A11029), validated by the company
Fibrillarin, validated by the company and cited in 158 research publications
p-CHK1 S317, validated by the company and cited in 243 research publications
CHK1, validated by the company and cited in 789 research publications
p-CHK2 T68, validated by the company and cited in 19 research publications
CHK2, knockout validated by the company and cited in 17 research publications
p-H2A.X S139 (γH2AX), validated by the company and cited in 131 research publications
β-Actin, validated by the company and cited in nearly 4000 research publications
p-AMPKα T172, validated by the company and cited in 2954 research publications
AMPKα, validated by the company and cited in 428 research publications
p-mTOR S2481, validated by the company and cited in 376 research publications
mTOR, validated by the company and cited in 1605 research publications
α-Tubulin, validated by the company and cited in 122 research publications
SLC25A51/MCART1, validated by the company and cited in Sci. Adv. 2020; 6 : eabe5310.
Vinculin, validated by the company (enhanced-validation strategy) and cited in 1937 research publications
VDAC, validated by the company and cited in 298 research publications
COX IV, validated by the company and cited in 124 research publications
OPA1, validated by the company and cited in 52 research publications
ATPB, validated by the company and cited in 229 research publications
Horseradish peroxidase-conjugated anti-rabbit secondary antibody, validated by the company and cited in 659 research publications
Horseradish peroxidase-conjugated anti-mouse secondary antibody, validated by the company and cited in 428 research publications

Eukaryotic cell lines

Policy information about cell lines and Sex and Gender in Research

Cell line source(s)	U2OS osteosarcoma (ATCC HTB-96, female), HeLa cervical carcinoma (ATCC CCL-2, female), BJ normal skin fibroblasts (ATCC CRL-2522, male), MRC5 normal lung fibroblasts (ATCC CCL-171), MEFs (ATCC CRL-2991), BJ 5ta hTERT-immortalized skin fibroblasts (ATCC CRL-4001, male), T98G glioblastoma multiforme (ATCC CRL-1690, male) and U87 MG likely glioblastoma multiforme (ATCC HTB-14, male) from ATCC.
Authentication	All cell lines were purchased from ATCC and the catalog numbers have been added in Methods. From the purchased date, cells have not been authenticated .
Mycoplasma contamination	All cell lines used in this study were regularly tested by PCR for mycoplasma contamination and were negative.
Commonly misidentified lines (See ICLAC register)	No commonly misidentified cell lines were used in this study.

Flow Cytometry

Plots

Confirm that:

☒ The axis labels state the marker and fluorochrome used (e.g. CD4-FITC).

☒ The axis scales are clearly visible. Include numbers along axes only for bottom left plot of group (a 'group' is an analysis of identical markers).

☒ All plots are contour plots with outliers or pseudocolor plots.

☒ A numerical value for number of cells or percentage (with statistics) is provided.

Methodology

Sample preparation	Cells were incubated with EdU for 30 min at 37 degrees C, trypsinized, washed with PBS and fixed with 70% ice-cold ethanol. Samples were stored at -20 degrees C before EdU was detected using the Click-iT EdU Alexa Fluor 647 Imaging Kit according to manufacturer's instructions. After washing with PBS(+), cells were stained with Hoechst 33342, washed again with PBS(+), resuspended in PBS and analysed on the flow cytometer.
Instrument	FACSVerse (Becton Dickinson)
Software	Data were collected using BD FACSuite V1.0.6 software and analysed using FlowJo_V10 software.
Cell population abundance	Full cell populations were analysed without post-sort fractions.
Gating strategy	All flow cytometry experiments were gated and analysed similarly. The same gates were used for the control and experimental conditions. Cell debris were gated out (FSC/SSC values below 50K and above 200K were considered cell debris).

☒ Tick this box to confirm that a figure exemplifying the gating strategy is provided in the Supplementary Information.
