# Supplementary material for: NAD+ regulates nucleotide metabolism and genomic DNA replication
Source: Nat Cell Biol. 2023 Nov 13;25(12):1774–86. doi: 10.1038/s41556-023-01280-z (PMC10709141; doi:10.1038/s41556-023-01280-z)

Figure 2e (left crops) and Extended Data Figure 2f (right crops)

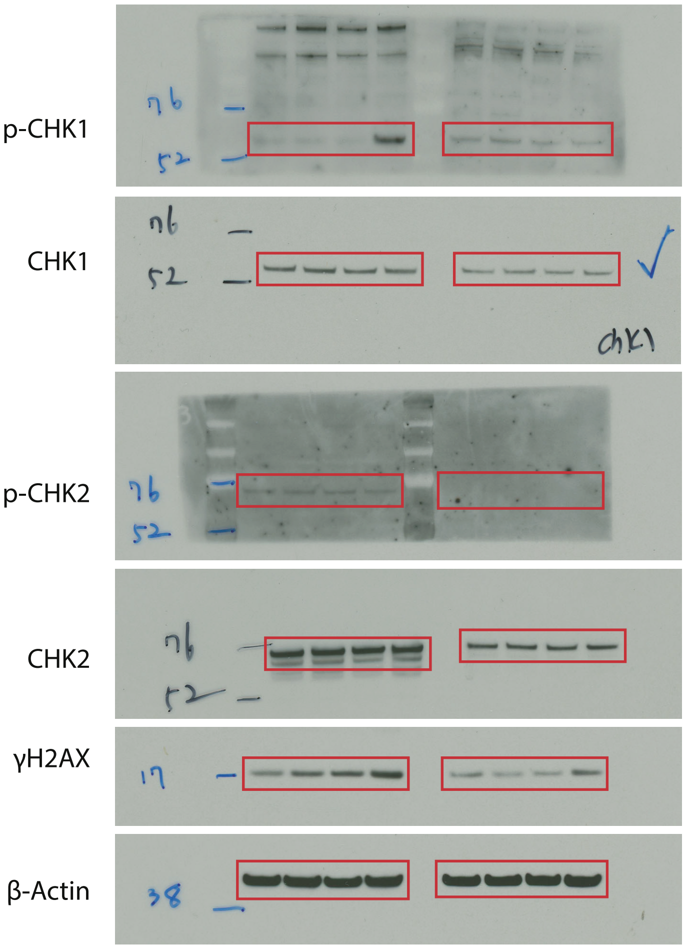

Extended Data Figure 2m

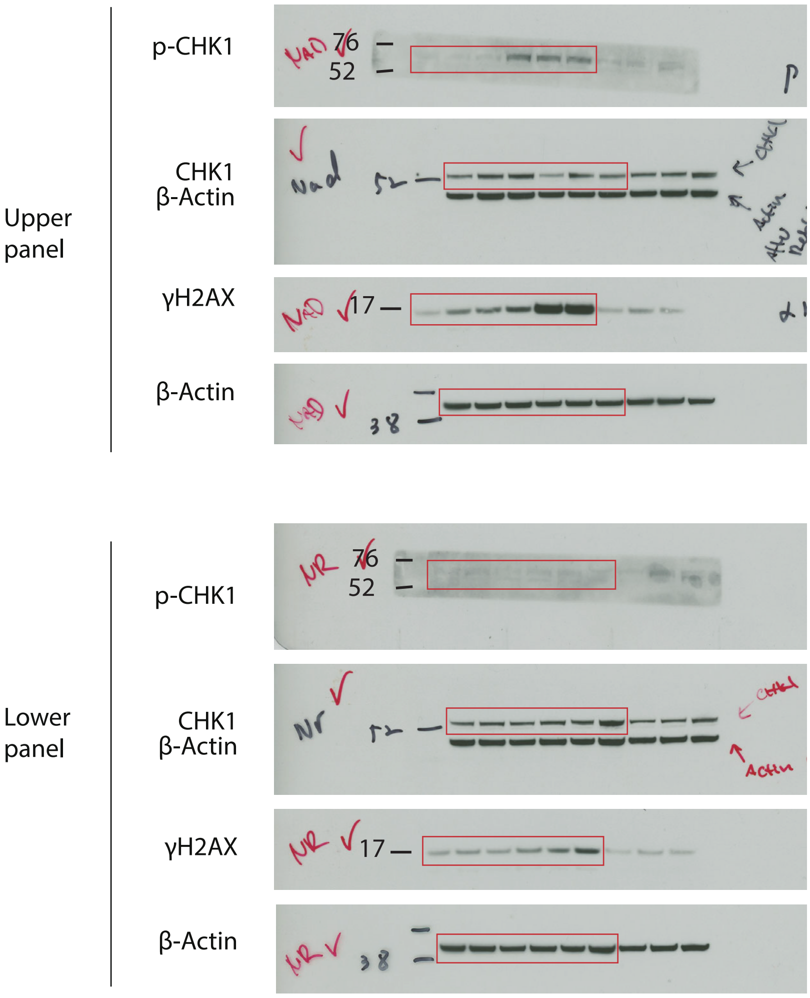

Extended Data Figure 3b

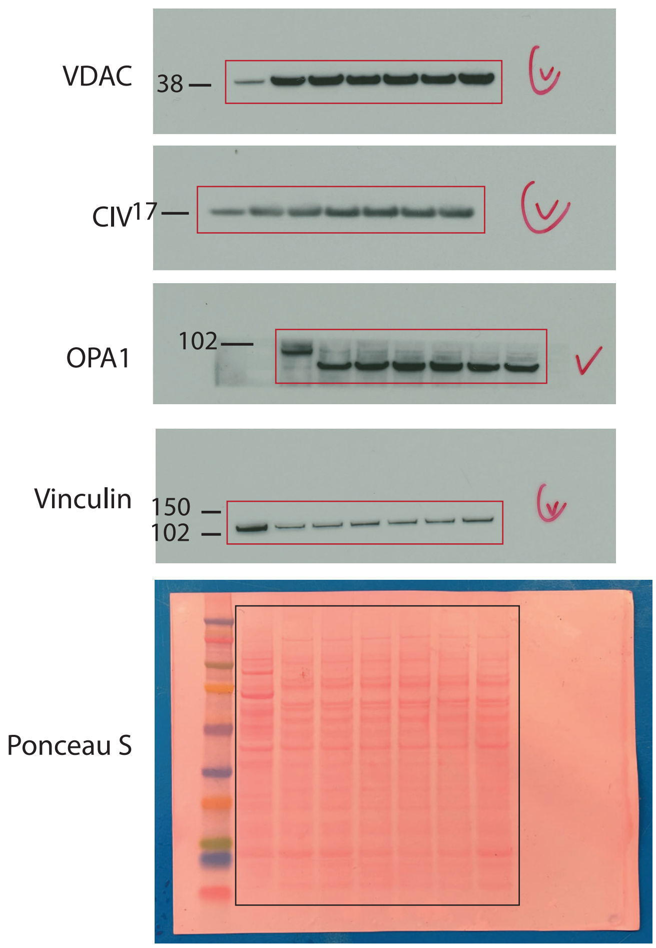

Extended Data Figure 6b

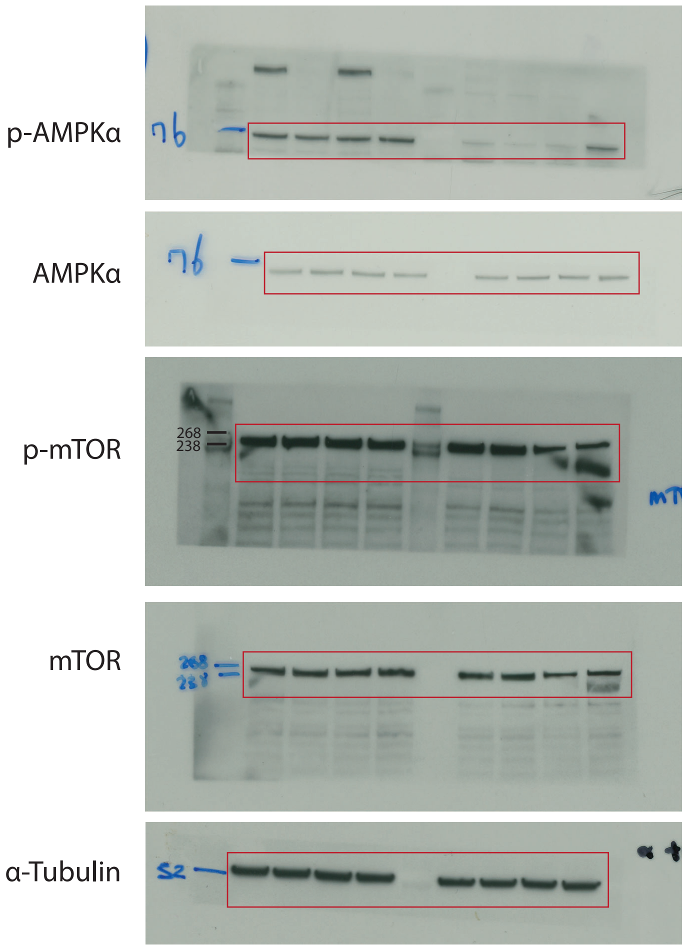

Extended Data Figure 9a

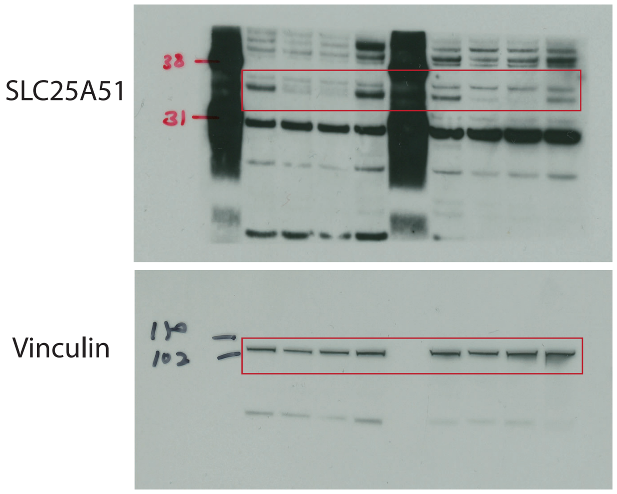

Extended Data Figure 9b (left crops) and 9c (right crops)

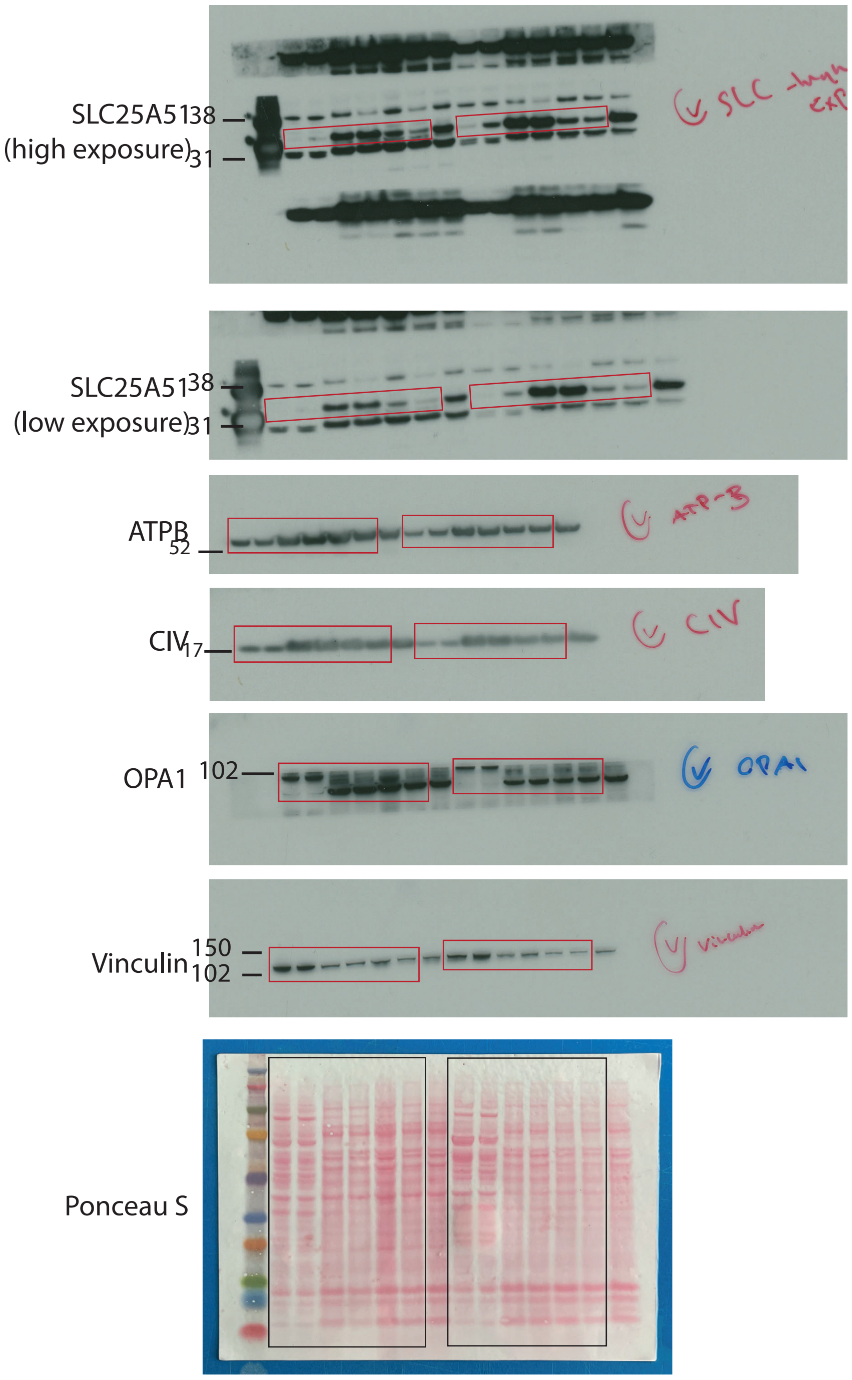

Supplement: Supplementary file 20 — Uncropped blot images of Fig. 2, and Extended Data Figs. 2, 3, 6 and 9. [file 41556_2023_1280_MOESM20_ESM.pdf]
